# Dobutamine Stress Echocardiography Ischemia as a Predictor of the Placebo-Controlled Efficacy of Percutaneous Coronary Intervention in Stable Coronary Artery Disease: The Stress Echocardiography–Stratified Analysis of ORBITA

**DOI:** 10.1161/CIRCULATIONAHA.119.042918

**Published:** 2019-11-11

**Authors:** Rasha K. Al-Lamee, Matthew J. Shun-Shin, James P. Howard, Alexandra N. Nowbar, Christopher Rajkumar, David Thompson, Sayan Sen, Sukhjinder Nijjer, Ricardo Petraco, John Davies, Thomas Keeble, Kare Tang, Iqbal Malik, Nina Bual, Christopher Cook, Yousif Ahmad, Henry Seligman, Andrew S.P. Sharp, Robert Gerber, Suneel Talwar, Ravi Assomull, Graham Cole, Niall G. Keenan, Gajen Kanaganayagam, Joban Sehmi, Roland Wensel, Frank E. Harrell, Jamil Mayet, Simon Thom, Justin E. Davies, Darrel P. Francis

**Affiliations:** 1 National Heart and Lung Institute, Imperial College London, UK (R.K.A-L., M.J.S.-S., J.P.H., A.N.N., C.R., D.T., S.S., S.N., R.P., I.M., C.C., Y.A., H.S., G.C., R.W., J.M., S. Thom, D.P.F.).; 2 Imperial College Healthcare NHS Trust, London, UK (R.K.A-L., M.J.S.-S., J.P.H., A.N.N., C.R., S.S., S.N., R.P., I.M., C.C., Y.A., H.S., R.A., G.C., G.K., J.M., J.E.D., D.P.F.).; 3 Essex Cardiothoracic Centre, Basildon, UK (J.D., T.K., K.T.).; 4 Anglia Ruskin University, Chelmsford, UK (J.D., T.K.).; 5 Cardiff Royal Infirmary, UK (A.S.P.S.).; 6 East Sussex Healthcare NHS Trust, Hastings, UK (R.G.).; 7 Royal Bournemouth and Christchurch NHS Trust, UK (S. Talwar).; 8 West Hertfordshire Hospitals NHS Trust, Watford, UK (N.G.K., J.S.).; 9 Vanderbilt University School of Medicine, Department of Biostatistics, Nashville, TN (F.E.H.).

**Keywords:** angina, stable, coronary artery disease, echocardiography, stress, percutaneous coronary intervention

## Abstract

**Background:**

Dobutamine stress echocardiography is widely used to test for ischemia in patients with stable coronary artery disease. In this analysis, we studied the ability of the prerandomization stress echocardiography score to predict the placebo-controlled efficacy of percutaneous coronary intervention (PCI) within the ORBITA trial (Objective Randomised Blinded Investigation With Optimal Medical Therapy of Angioplasty in Stable Angina).

**Methods:**

One hundred eighty-three patients underwent dobutamine stress echocardiography before randomization. The stress echocardiography score is broadly the number of segments abnormal at peak stress, with akinetic segments counting double and dyskinetic segments counting triple. The ability of prerandomization stress echocardiography to predict the placebo-controlled effect of PCI on response variables was tested by using regression modeling.

**Results:**

At prerandomization, the stress echocardiography score was 1.56±1.77 in the PCI arm (n=98) and 1.61±1.73 in the placebo arm (n=85). There was a detectable interaction between prerandomization stress echocardiography score and the effect of PCI on angina frequency score with a larger placebo-controlled effect in patients with the highest stress echocardiography score (*P*_interaction_=0.031). With our sample size, we were unable to detect an interaction between stress echocardiography score and any other patient-reported response variables: freedom from angina (*P*_interaction_=0.116), physical limitation (*P*_interaction_=0.461), quality of life (*P*_interaction_=0.689), EuroQOL 5 quality-of-life score (*P*_interaction_=0.789), or between stress echocardiography score and physician-assessed Canadian Cardiovascular Society angina class (*P*_interaction_=0.693), and treadmill exercise time (*P*_interaction_=0.426).

**Conclusions:**

The degree of ischemia assessed by dobutamine stress echocardiography predicts the placebo-controlled efficacy of PCI on patient-reported angina frequency. The greater the downstream stress echocardiography abnormality caused by a stenosis, the greater the reduction in symptoms from PCI.

**Clinical Trial Registration:**

URL: https://www.clinicaltrials.gov. Unique identifier: NCT02062593.

## Abstract 2

Supplemental Digital Content is available in the text.

**Clinical Perspective**

**What Is New?**

- This report of ORBITA (Objective Randomised Blinded Investigation With Optimal Medical Therapy of Angioplasty in Stable Angina), stratified by ischemia assessed by stress echocardiography, provides the first placebo-controlled evidence of an association between stress echocardiography ischemia and the magnitude of placebo-controlled benefit attributable to percutaneous coronary intervention.
- Prerandomization stress echocardiography score predicts the placebo-controlled effect of percutaneous coronary intervention on angina frequency score.

**What Are the Clinical Implications?**

- Although in ORBITA there was no detectable placebo-controlled reduction in angina frequency with percutaneous coronary intervention, this analysis shows that the greater the prerandomization stress echocardiography score, the greater the placebo-controlled reduction in angina.
- For patients with a stress echocardiography score of at least 1, there is a clear placebo-controlled reduction in patient-reported symptoms with percutaneous coronary intervention.
- This dependence of symptomatic relief on prerandomization ischemia was evident with stress echocardiography, but not with invasive physiology.

**Editorial, see p 1981**

The primary results of the ORBITA trial (Objective Randomised Blinded Investigation With Optimal Medical Therapy of Angioplasty in Stable Angina) showed a smaller-than-expected effect size of percutaneous coronary intervention (PCI) in comparison with placebo in single-vessel stable coronary artery disease on the primary end point of change in treadmill exercise time.^1^ These findings of ORBITA contrasted with those of previous unblinded trials which showed that patients aware that they had received PCI had a clear improvement in exercise time, reduction in angina, and improved quality of life, in comparison with patients aware that they had not received PCI.^2–7^

Although there was no significant difference between PCI and placebo groups in the patient-reported and physician-assessed symptom and quality-of-life end points, ischemia as assessed by dobutamine stress echocardiography (DSE) wall motion score index showed a clear reduction with PCI (prerandomization to follow-up increment: –0.07 in the PCI arm versus 0.02 in the placebo arm, *P*<0.0001).

Building on the primary analysis, the physiology-stratified analysis of ORBITA found that the severity of ischemia assessed by prerandomization fractional flow reserve (FFR) and instantaneous wave-free ratio (iFR) predicted the degree of improvement of ischemia as assessed by DSE score in the 196 patients with prerandomization invasive physiology data.^8^ However, there was no detectable interaction between invasive physiology and the placebo-controlled effect of PCI on symptoms or exercise time. Again, these findings contrasted with those of unblinded studies.^9,10^ Without placebo control, and with staff aware of measurements, there was a clear relationship between FFR or iFR and symptoms or exercise time.

In previous data sets^11^ and in ORBITA, PCI almost completely normalizes any left ventricular wall motion abnormalities detected by DSE. However, there has been no assessment of whether the magnitude of baseline stress echocardiography ischemia determines the magnitude of symptom relief from PCI, beyond any placebo effect.

In the present analysis of patients who had prerandomization DSE data, we stratified by the prerandomization stress echocardiography score and assessed its impact on the placebo-controlled effect of PCI on the primary and secondary end points of ORBITA.

## Methods

The data, analytical methods, and study materials will not be made available to other researchers for purposes of reproducing the results or replicating the procedure. The London Central Research Ethics Committee (reference 13/LO/1340) approved the study and written consent was obtained from all patients before their enrollment.

### Study Design

The design of the ORBITA trial has been reported previously.^1^ In brief, the ORBITA trial was a double-blind randomized, controlled trial comparing PCI with a placebo procedure in patients with stable angina and angiographically severe single-vessel coronary artery disease. Intensive medical therapy was given to both groups. Before randomization, patients had assessment of symptom and quality-of-life questionnaires, cardiopulmonary exercise testing using a smoothed modified Bruce protocol, DSE, and FFR and iFR measurement.

### Blinding and Randomization

Patients were randomly assigned 1:1 to PCI or a placebo procedure. Patients and the medical team outside the catheterization laboratory were blinded to treatment allocation as previously described.^1^

### Study End Points and Follow-Up

At the end of the 6-week blinded period, patients returned for the repeat of all prerandomization tests including symptom and quality-of-life questionnaires, cardiopulmonary exercise testing, and DSE.

### DSE Assessment

Patients were instructed to omit β-blockers for at least 24 hours before DSE. The test was performed by a physician and a sonographer. The patient, physician, and sonographer were all blinded to allocation arm.

Echocardiography was performed using contrast for all studies. The contrast agent used was a commercially available sulfur hexafluoride microbubble preparation, SonoVue (Bracco Imaging SpA). This agent was administered in 0.3-mL bolus doses intravenously for each image acquisition followed by 1 to 2 mL of saline flush. After acquisition of resting images to exclude significant valvular disease, intravenous dobutamine was infused at a starting dose of 10 µg·kg^–1^·min^–1^ followed by increasing doses of 20 µg·kg^–1^·min^–1^, 30 µg·kg^–1^·min^–1^, up to a maximum of 40 µg·kg^–1^·min^–1^ in 3-minute stages. Intravenous atropine was administered in 300-μg boluses up to a maximum of 1200 μg for those patients not achieving 85% of the predicted maximal heart rate. Images were taken in the apical 2-chamber, 3-chamber, 4-chamber, and parasternal short-axis views at baseline, low-dose stress, high-dose stress, and recovery.

### DSE Reporting

Analysis was also performed with reporters blinded to treatment allocation and phase (prerandomization or follow-up), using an online reporting tool.

Each scan was examined twice by 6 imaging consultants (R.A., D.P.F., G.C., G.K., J.S., and N.G.K.) who were blinded to treatment allocation, time point of the scan, their colleagues’ opinions, and their own first opinion.

Stress echocardiography results are presented in a manner that represents the number of hypokinetic segments (with akinetic segments scoring double, dyskinetic segments scoring triple, and aneurysmal segments scoring quadruple). The left ventricle was divided into the standard 17-segment model. Wall motion was scored as follows: normal=0, hypokinetic=1, akinetic=2, dyskinetic=3, or aneurysmal=4. Individual wall abnormality scores at peak stress were summed. Both opinions from all 6 consultants were averaged. The stress echocardiography score^8^ can be broadly converted to classical wall motion score index as follows: wall motion score index=1+(stress echocardiography score)/17. More details are provided in the online-only Data Supplement.

### Statistical Analysis

Summary statistics were presented as appropriate for baseline characteristics. To assess the observer variability of the stress echocardiography score, we calculated the mean inter- and intraobserver absolute differences.^12^

Models were fitted for each end point. Models using ordinary least squares were used for the continuous variables: Seattle Angina Questionnaire (SAQ) physical limitation and quality-of-life score, EuroQOL 5 (EQ-5D-5L) descriptive score, and exercise time. Proportional odds logistic models were used for ordinal variables: SAQ angina frequency score and freedom from angina, and Canadian Cardiovascular Society angina class. For each of the components of the SAQ, and freedom from angina, as well, a higher score represents a better health state; therefore, an odds ratio >1 suggests that a better health state was achieved with PCI over placebo.

To assess the interaction of prerandomization stress echocardiography score with each continuous and categorical outcome variable, the follow-up value was modeled conditioned on the prerandomization value transformed by a restricted cubic spline with 3 parameters, and randomization arm. A model was then fitted with prerandomization stress echocardiography score interacting with the randomization arm with a restricted cubic spline with 3 parameters. Knots were placed at the standard positions of 25th, 50th, and 75th percentiles of the covariate distribution. Therefore, the shape of the effect was allowed to vary over treatments.^13^ Graphs are shown of the end points against prerandomization stress echocardiography score. The contrast between the arms was generated with an adjustment for the median value of the prerandomization value. The vertical coordinate of the graphs is the difference in end value between the 2 arms, conditioned on their prerandomization value. We report the interaction with treatment as the *P* value (*P*_interaction_) from the combined main effect and interaction effect.

Analyses were performed using the open-source statistical environment R,^14^ with the package rms for regression modeling^15^ and ggplot2 for graphs.^16^

## Results

Prerandomization stress echocardiography scores were available for 183 patients (98 PCI and 85 placebo) of the 200 patients randomly assigned in ORBITA. Of the remaining 17 patients, 1 had poor-quality echocardiographic imaging windows, 6 had a previous adverse reaction to dobutamine (5 minor but limiting reactions, 1 severe life-threatening reaction), 6 had a clinical contraindication to dobutamine administration, and in 4 there were logistical reasons as to why the test was not performed.

### Patient Demographics

Table 1 shows the patient demographic data. The majority of patients had normal left ventricular systolic function (94.9% in the PCI arm and 90.6% in the placebo arm). Median angina duration was 5 months in the PCI arm (interquartile range, 4–10) and 6 months in the placebo arm (interquartile range, 4–9).

**Table 1.**
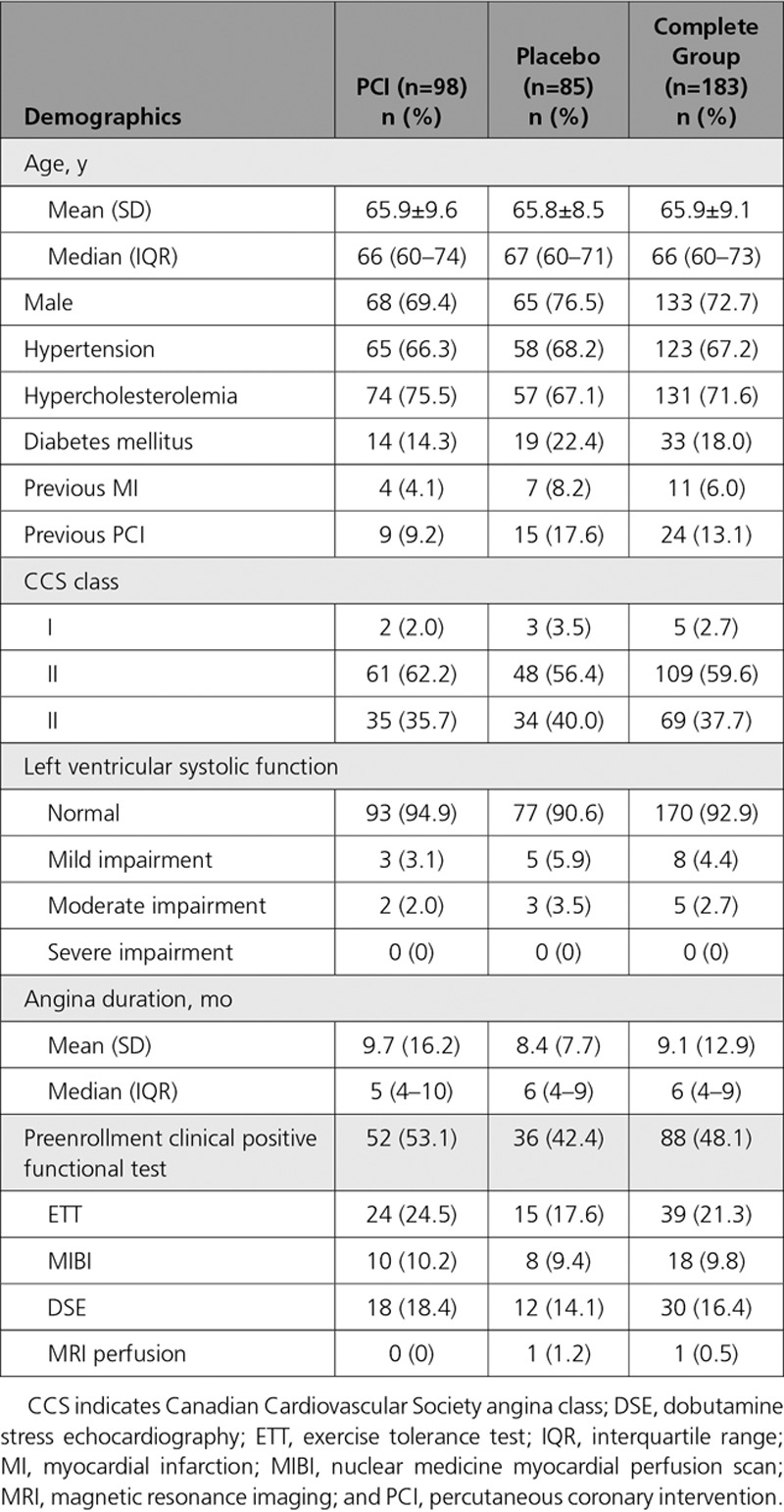
Patient Demographics at Enrollment

### Procedural Demographics

Table 2 shows the procedural demographic data. The majority of lesions were in the left anterior descending artery (68.4% PCI and 69.4% placebo); 16.3% (16/98) patients in the PCI arm and 11.8% (10/85) patients in the placebo arm had serial lesions in a single coronary artery. The mean diameter stenosis by quantitative coronary angiography was 64.3±13.9% in the PCI arm and 64.1±13.4% in the placebo arm. The mean prerandomization stress echocardiography score was 1.56±1.77 in the PCI arm and 1.61±1.73 in the placebo arm. The distribution of prerandomization stress echocardiography scores is shown in Figure I in the online-only Data Supplement. The prerandomization mean stress wall motion score for each segment as associated with target vessel coronary territory is shown in Table I in the online-only Data Supplement. The mean inter- and intraobserver absolute differences of the stress echocardiography score were 1.4 and 1.0 stress echocardiography units, respectively. The mean FFR was 0.69±0.16 for the PCI arm and 0.69±0.16 for the placebo arm, and the mean iFR was 0.76±0.22 for the PCI arm and 0.77±0.20 for the placebo arm. After intervention with drug-eluting stents implanted in the PCI arm, the mean FFR increased to 0.90±0.05 and iFR increased to 0.95±0.04. The change in stress echocardiography score from prerandomization to follow-up in 161 patients with stress echocardiography data at both time points is shown in Figure II in the online-only Data Supplement.

**Table 2.**
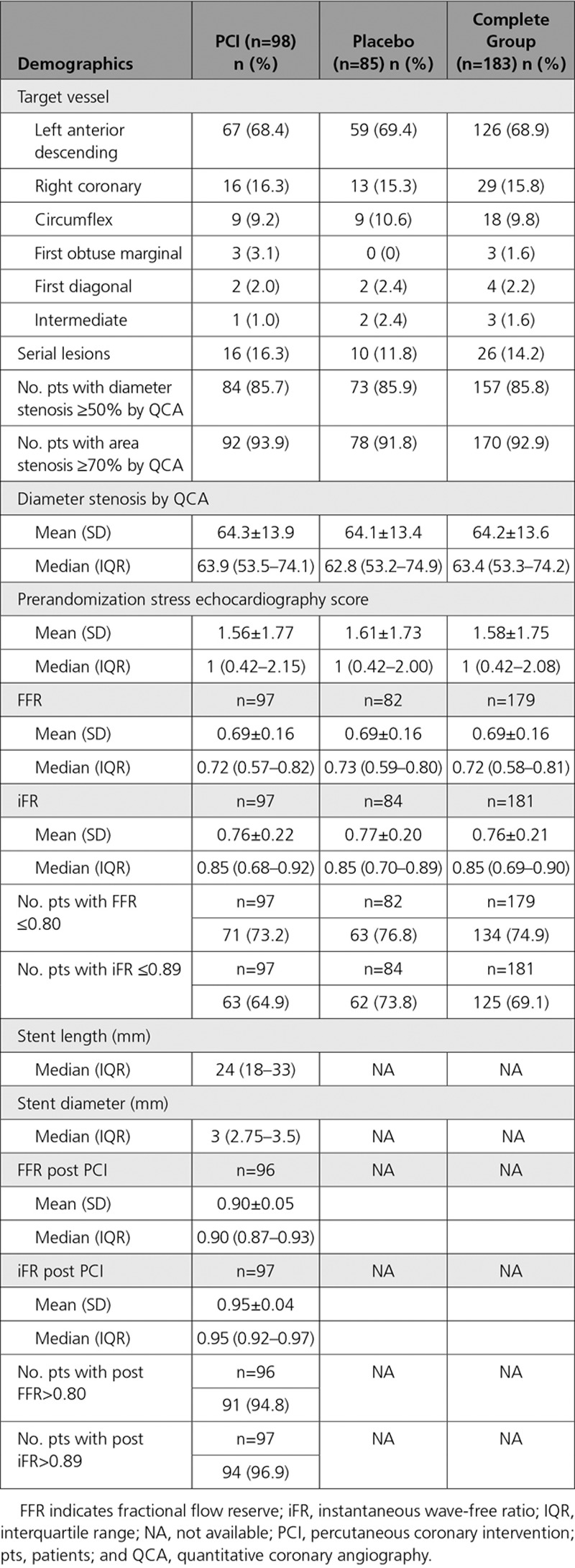
Procedural Demographics

### Relationship Between FFR and iFR and Stress Echocardiography Score

**Figure 1.**
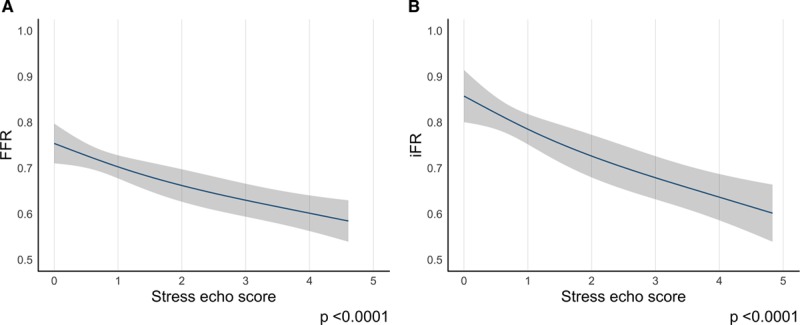
**Relationship between prerandomization stress echocardiography score and prerandomization FFR and iFR. A**, Relationship between prerandomization stress echocardiography score and prerandomization FFR. **B**, Relationship between prerandomization stress echocardiography score and prerandomization iFR. echo indicates echocardiography; FFR, fractional flow reserve; and iFR, instantaneous wave-free ratio.

Prerandomization FFR or iFR and DSE data were available in 179 patients and 181 patients, respectively (in 2 patients we were unable to elicit a hyperemic response to adenosine and, therefore, only iFR data are available). Figure 1A shows the relationship between prerandomization FFR and prerandomization stress echocardiography score. As the stress echocardiography score became larger with a greater number of ischemic myocardial segments, the FFR value decreased, therefore showing a greater degree of ischemia (*P*_correlation_<0.0001). At a stress echocardiography score of 0 (normal), the mean FFR was 0.76±0.17 (n=16). For scores intermediate between 0 and 1, mean FFR was 0.72±0.14 (n=72); at ≥1 to <2, 0.71±0.12 (n=45); at ≥2 to <3, 0.65±0.17 (n=21); and at ≥3, 0.55±0.18 (n=25). Figure 1B shows the relationship between prerandomization iFR and prerandomization stress echocardiography score. Similarly, as the stress echocardiography score became larger with a greater number of ischemic myocardial segments, the iFR also decreased, showing a greater degree of ischemia (*P*<0.0001). At a stress echocardiography score of 0 (normal), the mean iFR was 0.85±0.16 (n=16). For scores intermediate between 0 and 1, mean iFR was 0.82±0.16 (n=73); at ≥1 to <2, 0.80±0.16 (n=45); at ≥2 to <3, 0.67±0.26 (n=21); and at ≥3, 0.57±0.27 (n=26).

### Patient-Reported Symptoms

#### SAQ Angina Frequency Score and Freedom From Angina

**Figure 2.**
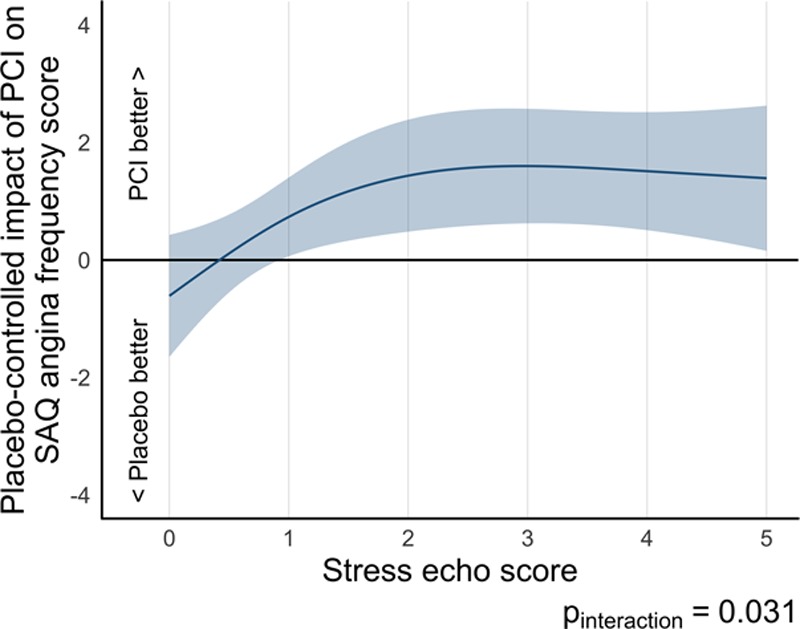
**Relationship of treatment difference in Seattle Angina Questionnaire (SAQ) angina frequency score at follow-up to prerandomization stress echocardiography score by randomization arm.** There is a significant interaction between stress echocardiography score and Seattle Angina Frequency score with a progressive tendency for larger effects on angina frequency score with higher stress echocardiography score (*P*_interaction_=0.031). echo indicates echocardiography; and PCI, percutaneous coronary intervention.

Paired SAQ angina frequency data were available for 176 patients in the stress echocardiography–stratified analysis of ORBITA (96 in the PCI arm and 80 in the placebo arm). Overall, there was little evidence that PCI improved angina frequency score more than placebo (odds ratio [OR], 1.68 [95% CI, 0.96–2.95], *P*=0.069) in this DSE subset (Table 3). However, there was a detectable interaction between prerandomization stress echocardiography score and the effect of PCI on angina frequency score with a larger placebo-controlled effect of PCI in patients with the highest stress echocardiography score (*P*_interaction_=0.031; Figure 2). This interaction resulted in patients with a prerandomization stress echocardiography score of ≥1 being more likely to have a lower angina frequency score with PCI than with placebo (OR, 3.18 [95% CI, 1.38–7.34], *P*=0.007; Table II in the online-only Data Supplement).

**Table 3.**
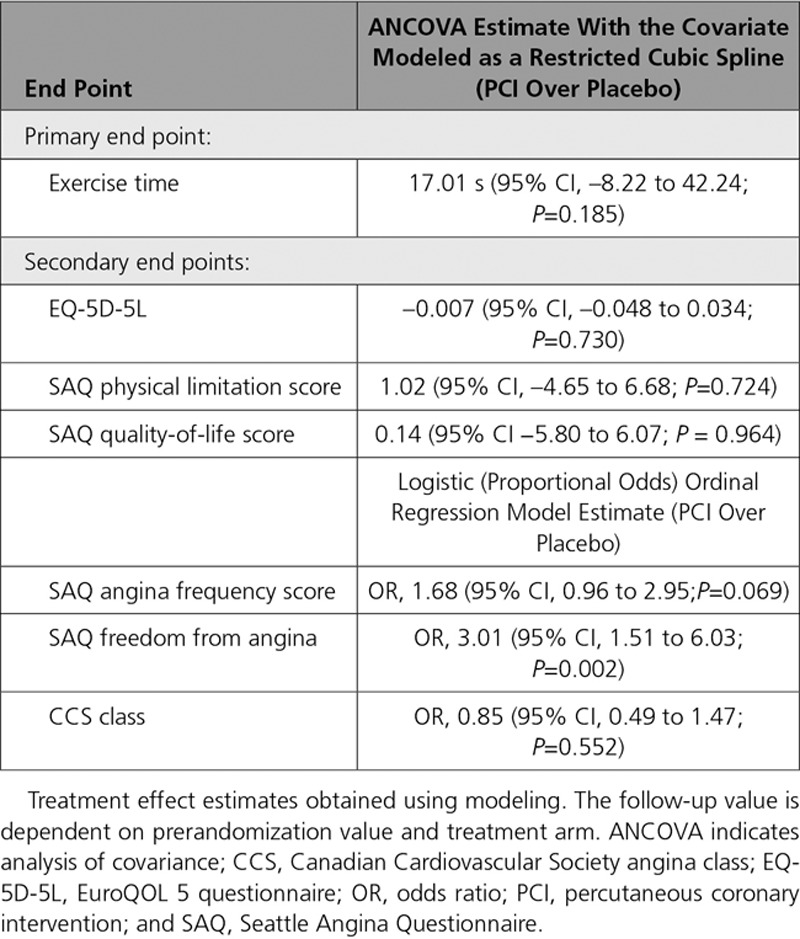
End Point Analysis

**Figure 3.**
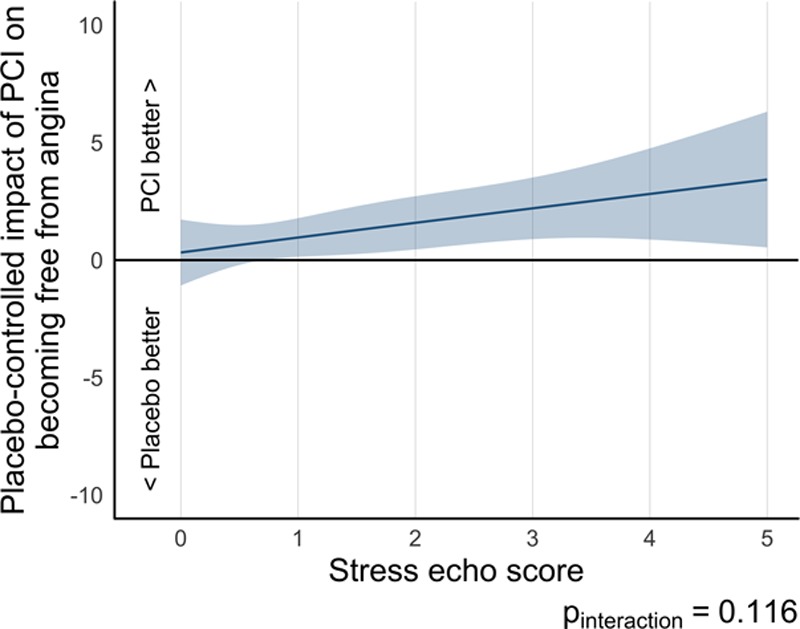
**Relationship of treatment difference in freedom from angina and prerandomization stress echocardiography by randomization arm.** There is no discernible dependence on prerandomization stress echocardiography score. echo indicates echocardiography; and PCI, percutaneous coronary intervention.

Paired angina freedom data were available for 175 patients in the stress echocardiography–stratified analysis of the ORBITA (95 in the PCI arm and 80 in the placebo arm). PCI was more likely to result in patient-reported freedom from angina than placebo (OR, 3.01 [95% CI, 1.51–6.03], *P*=0.002) in this DSE subset (Table 3). There was no detectable interaction between prerandomization stress echocardiography score and the effect of PCI on freedom from angina (*P*_interaction_=0.116; Figure 3). Patients with a prerandomization stress echocardiography score of ≥1 were more likely to be free from angina with PCI than with placebo (OR, 4.62 [95% CI, 1.70–12.60], *P*=0.003; Table III in the online-only Data Supplement).

#### SAQ Physical Limitation Score and Quality-of-Life Score and EQ-5D-5L Score

**Figure 4.**
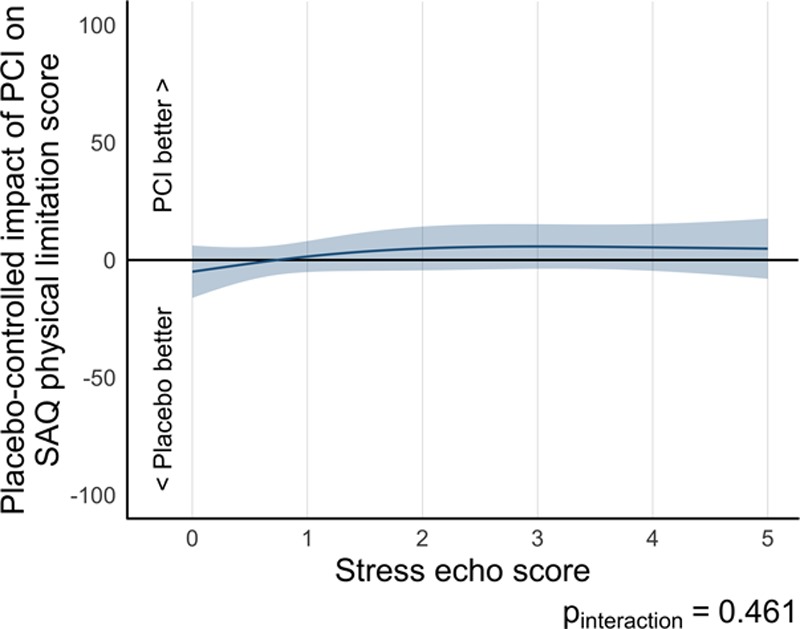
**Relationship of treatment difference in Seattle Angina Questionnaire (SAQ) physical limitation score and prerandomization stress echocardiography by randomization arm.** There is no discernible dependence on prerandomization stress echocardiography score. echo indicates echocardiography; and PCI, percutaneous coronary intervention.

Paired SAQ physical limitation data, SAQ quality of life, and EQ-5D-5L data were available for 171 patients (93 in the PCI arm and 78 in the placebo arm), 175 patients (96 in the PCI arm and 79 in the placebo arm), and 175 patients (96 in the PCI arm and 79 in the placebo arm), respectively, in the stress echocardiography–stratified analysis of the ORBITA. There was no evidence that PCI improved physical limitation score more than placebo (1.02 [95% CI, –4.65 to 6.68], *P*=0.724 in this DSE subset, quality-of-life score more than placebo (0.14 [95% CI, –5.80 to 6.07], *P*=0.964 in this DSE subset or EQ-5D-5L quality-of-life score more than placebo (–0.007 [95% CI, –0.048 to 0.034], *P*=0.73; Table 3). There was no detectable interaction between prerandomization stress echocardiography score and the effect of PCI on physical limitation score (*P*_interaction_=0.461; Figure 4), quality-of-life score (*P*_interaction_=0.689; Figure III in the online-only Data Supplement) or quality of life as assessed by EQ-5D-5L (*P*_interaction_=0.789; Figure IV in the online-only Data Supplement).

### Physician-Assessed Symptoms

Paired Canadian Cardiovascular Society class data were available for 179 patients (98 in the PCI arm and 81 in the placebo arm). There was no evidence that PCI improved Canadian Cardiovascular Society class more than placebo (OR, 0.85 [95% CI, 0.49–1.47], *P*=0.552; Table 3). There was no detectable interaction between prerandomization stress echocardiography score and the effect of PCI on Canadian Cardiovascular Society class (*P*_interaction_=0.693; Figure V in the online-only Data Supplement).

### Exercise Time

**Figure 5.**
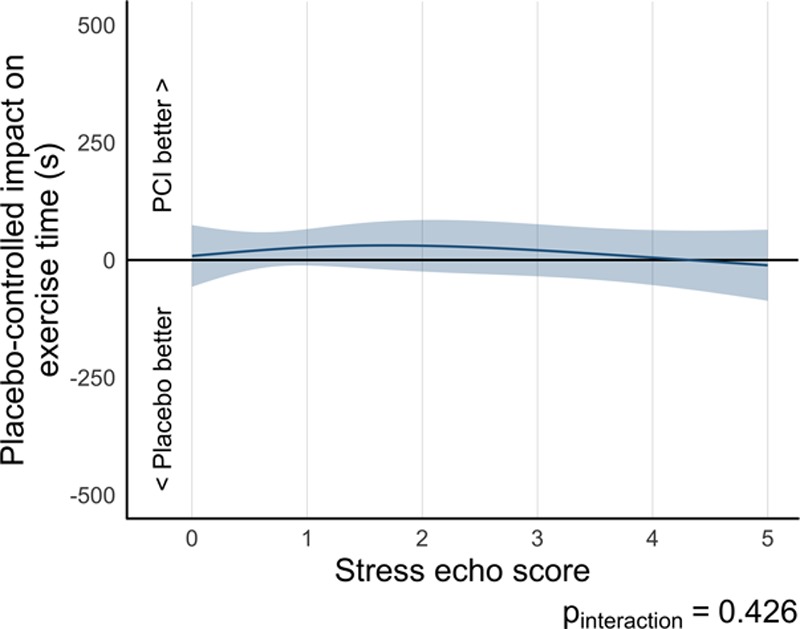
**Relationship of treatment difference in exercise time and prerandomization stress echocardiography by randomization arm.** There is no discernible dependence on prerandomization stress echocardiography score. echo indicates echocardiography; and PCI, percutaneous coronary intervention.

Paired exercise time data were available for 177 patients (97 in the PCI arm and 80 in the placebo arm). The estimated effect of PCI over placebo on exercise time using regression modeling was 17.0 seconds (95% CI, –8.22 to 42.2; *P*=0.19) in this DSE subset (Table 3). There was no detectable interaction between prerandomization stress echocardiography score and the effect of PCI on exercise time (*P*_interaction_=0.426; Figure 5). There was no evidence that patients with a prerandomization stress echocardiography score of ≥1 were more likely to have more exercise time improvement with PCI that with placebo (18.4 seconds [95% CI, –18.3 to 55.1], *P*=0.322; Table IV in the online-only Data Supplement).

## Discussion

This is the first placebo-controlled analysis of the relationship between ischemia assessed by DSE and the efficacy of PCI in stable coronary artery disease. The prerandomization stress echocardiography score significantly predicted the placebo-controlled impact of PCI on patient-reported angina frequency. The greater the ischemia, the greater the symptom improvement. Second, although a greater proportion of patients became free from angina in the PCI arm than in the placebo arm, there was no evidence of interaction between this effect and the prerandomization stress echocardiography score. Finally, there was strong correlation between prerandomization stress echocardiography score and invasive physiology measured by FFR and iFR. The greater the number of ischemic regional wall segments, the lower the FFR and iFR.

**Figure 6.**
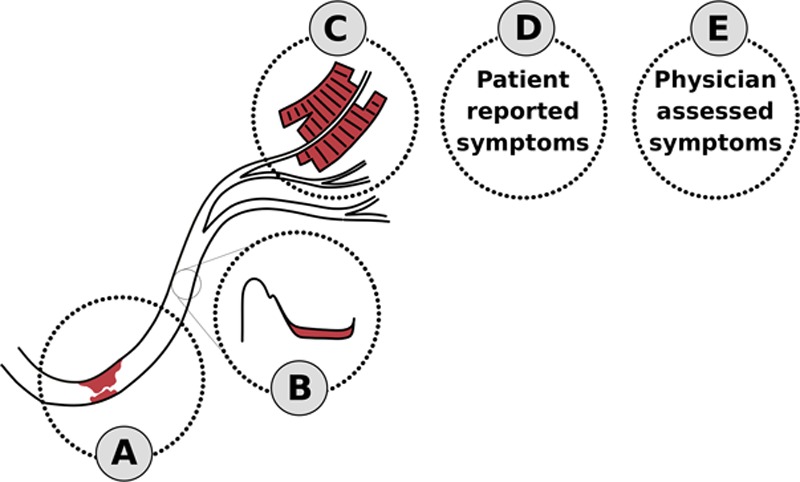
**A proposed sequence of steps in the pathway of ischemia.** Coronary stenosis (step A) causes coronary hemodynamic insufficiency (step B) which leads to stress-induced myocardial ischemia. This manifests as wall motion abnormalities on imaging tests (step C) and causes pain that is verbalized by the patient (step D) and recorded by the physician (step E). The magnitude of association between measurements is likely to be stronger between adjacent steps than steps further apart.

We propose an explanation for these and previously reported results from the ORBITA trial.^1,8^ The progressive decline in strengths of association may result from the sequence of steps in the pathway of ischemia, with the signal becoming increasingly dilute at later steps in the chain (Figure 6). PCI immediately relieves the angiographic stenosis (step A). As a result, the intracoronary physiology improves (step B). This in turn can reduce myocardial ischemia, resolving wall motion abnormalities (step C). Angina, which is presumably a sensation arising from ischemia, can be alleviated by this and reported by the patient (step D). The physician, who relies on the patient’s verbal and nonverbal cues, is one step further removed (step E).

In the primary analysis of ORBITA,^1^ PCI had an extremely clear effect on anatomy (step A, *P*< 0.001 for anatomical stenosis). There was a very clear effect on physiology (step B, *P*< 0.001 for FFR and *P*< 0.001 for iFR). The effect on myocardial wall motion abnormality was still clear (step C, *P*< 0.001). One step further, and there was no longer a clear effect on angina (step D), with the preplanned analysis of SAQ angina frequency showing no detectable change (*P*=0.260) and a post hoc analysis showing a clearer effect on the dichotomous end point of freedom from angina (*P*=0.006).^8^

Although the relatively weak effects on steps D to E in Figure 6 were a surprise in the context of extensive previous experience,^2,7,17,18^ the previous experience was unblinded. Clinical staff are trained to interpret the information in steps A, B, and C and explain to the patient that the problem has been resolved. Therefore, the impact of reassurance alone and simply being told that their lesion was not flow-limiting dramatically reduced angina rates from 88% to 54% in the DEFER trial (Deferral of PTCA Versus Performance of PTCA) and 64% to 15% in the FAME-2 trial (Fractional Flow Reserve-Guided Percutaneous Coronary Intervention Plus Optimal Medical Treatment Versus Optimal Medical Treatment Alone in Patients With Stable Coronary Artery Disease).^17,19^ An unblinded PCI procedure gives this reassurance, that there is now no significant lesion, but also gives patients an expectation that the symptoms were attributable to the treated lesion and should now resolve. Because of this powerful reassurance effect, it is not possible to gauge how much of the symptom relief from unblinded PCI is purely attributable to the physiological effect of stenosis relief^20–22^ and how much may be attributable to the placebo component of unblinded PCI.^7,18^

This stress echocardiography–stratified analysis shows the link between stress-induced myocardial wall motion abnormalities (step C) and patient-reported angina frequency (step D). The greater the ischemia on DSE, the greater the placebo-controlled angina relief from PCI.

The invasive physiology measures, FFR and iFR, are further upstream (step B). This may explain why they were not as strongly associated as DSE with the magnitude of placebo-controlled angina relief from PCI.^8^ Physiological features other than the transstenotic pressure gradient may affect whether the myocardium experiences sufficient ischemia to manifest stress echocardiography abnormalities or symptoms. For example, there may be microvascular dysfunction or differential sensitivity of the myocardium to intracoronary pressure. Many patients with obstructive epicardial stenoses also have microvascular disease. ORBITA did not acquire coronary microvascular function to distinguish between the various possibilities.

Another alternative proposed explanation might be that the conventional cut points for ischemia with FFR and iFR may not correspond to those of stress echocardiography. However, the threshold of FFR was defined by reference to tests such as stress echocardiography.^23^ Moreover, the differences in sensitivity/specificity cannot be the explanation, because, in both our FFR/iFR-stratified analysis^8^ and our present stress echocardiography–stratified analysis, the variables were treated continuously across their entire spectrum and not as a mere dichotomy.^24^

In ORBITA the research stress echocardiography (and the previously reported research FFR/iFR) were performed after the clinical decision to revascularize. These research assessments were not made available to the clinicians. Therefore, as might be expected in a trial of single-vessel coronary stenoses, many patients had a low stress echocardiography score. Nevertheless, there was not only a clear reduction of stress echocardiography score by PCI, but also a relationship between the prerandomization stress echocardiography score and the degree of angina relief beyond placebo from PCI.

PCI is known to improve ischemia as assessed by DSE.^1,11^ The COURAGE trial (Clinical Outcomes Utilizing Revascularization and Aggressive Drug Evaluation), which confirmed that PCI reduced ischemia on myocardial perfusion scans,^25^ showed that the baseline extent of ischemia did not predict the efficacy of PCI in reducing death or myocardial infarction.^26^ No previous studies have assessed the impact of prerandomization noninvasive ischemia on the placebo-controlled efficacy of PCI on symptom relief. The present analysis shows that the greater the degree of stress echocardiography ischemia preintervention, the greater the angina relief from PCI beyond placebo.

### Limitations of This Study

This analysis addresses only the 183 patients from the 200-patient ORBITA trial with prerandomization DSE. There is potential for bias if the remaining 17 patients differed in some way.

Our original expectation had been of a large PCI effect on exercise time. Because this expectation was not realized, there is reduced power to detect variation in exercise time effect across different prerandomization strata. Despite this, there was still a surprisingly clear relationship between prerandomization DSE and placebo-controlled angina relief.

The follow-up period may be considered short at 6 weeks. However, the effect of PCI on both angiographic and physiological improvement of a stenosis is immediate, and the primary results of ORBITA showed virtually complete normalization of stress echocardiography (assessed blinded to time point) at the 6-week follow-up scan. In previous trials, angina relief was seen a month post-PCI.^2^ Therefore, we believe that we should not regard 6-week data as premature.

Stress echocardiography assessment is known to have interobserver^27^ and intraobserver variability.^28^ To reduce the impact of this variability, each scan was reported twice by 6 different operators who were each blinded to the treatment allocation and time point of the scan and to their own and each other’s opinions. Each scan was therefore summarized as the mean of 12 opinions.

### Conclusions

Stratification of the primary and secondary end points of ORBITA by prerandomization DSE showed that the higher the stress echocardiography score, the greater the placebo-controlled efficacy of PCI on improvement in patient-reported frequency of angina.

In ORBITA, the effect of PCI was progressively less clear at each step in the chain from anatomy, to invasive hemodynamics, to stress echocardiography ischemia, and then to the frequency of angina.

We have previously found that there is a clear relationship between invasive physiology and stress echocardiography score, but no relationship between invasive physiology and placebo-controlled symptom improvement. The present analysis shows that there is clear evidence of a relationship between ischemia on stress echocardiography and the placebo-controlled efficacy of PCI on the frequency of angina.

## Acknowledgments

ORBITA was an investigator-led trial sponsored by Imperial College London. We thank our patients and their families for their dedication and support for the ORBITA trial. Special thanks to N. Bual for performing the stress echocardiography investigations. We thank the research and administrative teams at Imperial College Healthcare NHS Trust, Essex Cardiothoracic Centre, East Sussex Healthcare NHS Trust, Royal Devon and Exeter NHS Trust, and Royal Bournemouth and Christchurch NHS Trust for their dedication and support.

## Sources of Funding

The trial was funded by grants from National Institute for Health Research (NIHR) Imperial Biomedical Research Centre, Foundation for Circulatory Health, and Imperial College Healthcare Charity. Dr Howard is a PhD Training Fellow at the Wellcome Trust. Philips Volcano supplied the coronary pressure wires. We acknowledge the support of the NIHR Clinical Research Network (NIHR CRN).

## Disclosures

Drs J. E. Davies and Mayet hold patents pertaining to the iFR technology. Drs J. E. Davies and Sharp are consultants for Philips Volcano. Drs Al-Lamee, Sen, Petraco, Cook, and Nijjer have received speaker’s honoraria from Philips Volcano. Drs J. E. Davies and Keeble have received research grants from Philips Volcano. The other authors report no conflicts.

## Supplementary Material
