# Supplementary material for: Dobutamine Stress Echocardiography Ischemia as a Predictor of the Placebo-Controlled Efficacy of Percutaneous Coronary Intervention in Stable Coronary Artery Disease: The Stress Echocardiography–Stratified Analysis of ORBITA
Source: Circulation. 2019 Nov 11;140(24):1971–80. doi: 10.1161/CIRCULATIONAHA.119.042918 (PMC6903430; doi:10.1161/CIRCULATIONAHA.119.042918)

SUPPLEMENTAL MATERIAL

Dobutamine stress echo as a predictor of the placebo-controlled response to percutaneous coronary intervention in stable single vessel coronary artery disease: the stress echo-stratified analysis of ORBITA

Supplemental Methods

Dobutamine Stress Echocardiography

Rest and stress cardiac regional wall motion were assessed by using dobutamine stress echocardiography. The test was per- formed by a physician and sonographer. The patient, physician, and sonographer were all blinded to allocation arm.

Analysis was also performed blinded to treatment allocation and phase (pre-randomization or follow-up), using an online reporting tool. In the original ORBITA publication, analysis had been performed by 2 imaging consultants (R.A. and D.F.).

For the present physiology-stratified analysis of ORBITA, each scan received 12 opinions. Each scan was examined twice by 6 imaging consultants (R.A., D.F., G.C., G.K., J.S., and N.K.) who were blinded to treatment allocation, time point of the scan, their colleagues' opinions, and (on the second viewing) their own first opinion.

In this physiology-stratified analysis of ORBITA, for ease of reader interpretation, stress echocardiography results are presented in a manner that represents the number of hypokinetic segments (with akinetic segments scoring double, dyskinetic scoring triple, and aneurysmal segments scoring quadruple). In detail, the left ventricle was divided into the standard 17 segments. Wall motion was scored as follows: normal=0, hypokinetic=1, akinetic=2, dyskinetic=3, or aneurysmal=4. These individual wall abnormality scores at peak stress were summed. Both opinions from all 6 consultants were then averaged. This stress echo score can be broadly converted to classical wall motion score index as follows: wall motion score index=1+(stress echo score)/17.

Supplemental Tables

Supplemental Table 1
Pre-randomization mean stress wall motion score for each segment as associated with target vessel coronary territory

Myocardial segment	Coronary territory of target vessel		
	LAD n=133	Cx n=21	RCA n=29
**1**	0.042	0.004	0.032
**2**	0.042	0.008	0.020
**3**	0.031	0.032	0.066
**4**	0.035	0.067	0.124
**5**	0.046	0.103	0.224
**6**	0.003	0.012	0.009
**7**	0.173	0.028	0.118
**8**	0.112	0.020	0.069
**9**	0.061	0.056	0.072
**10**	0.050	0.032	0.095
**11**	0.055	0.060	0.144
**12**	0.029	0.012	0.078
**13**	0.214	0.036	0.129
**14**	0.127	0.044	0.101
**15**	0.142	0.044	0.069
**16**	0.208	0.083	0.170
**17**	0.315	0.143	0.187

Shaded light grey corresponds to the typical myocardial segments that are ascribed to each coronary artery territory. LAD territory includes the first diagonal. Cx territory includes first obtuse marginal/intermediate.

Supplemental Table 2
Angina frequency score stratified by stress echo score for complete group

Stress echo score < 1				Stress echo score ≥ 1			
n	OR	95% CI	p value	n	OR	CI	p value
88	0.97	0.45 to 2.12	0.945	88	3.18	1.38 to 7.34	0.007

Supplemental Table 3
Freedom from angina stratified by stress echo score for complete group

Stress echo score < 1				Stress echo score ≥ 1			
n	OR	95% CI	p value	n	OR	CI	p value
88	1.98	0.75 to 5.21	0.167	87	4.62	1.70 to 12.60	0.003

Supplemental Table 4
Exercise time stratified by stress echo score for complete group

Stress echo score < 1				Stress echo score ≥ 1			
n	Point Estimate (sec)	95% CI	p value	n	Point estimate (sec)	CI	p value
87	18.9	-16.9 to 54.7	0.298	84	18.4	-18.3 to 55.1	0.322

Supplemental Figures

Supplemental Figure 1
Distribution of pre-randomization stress echo scores

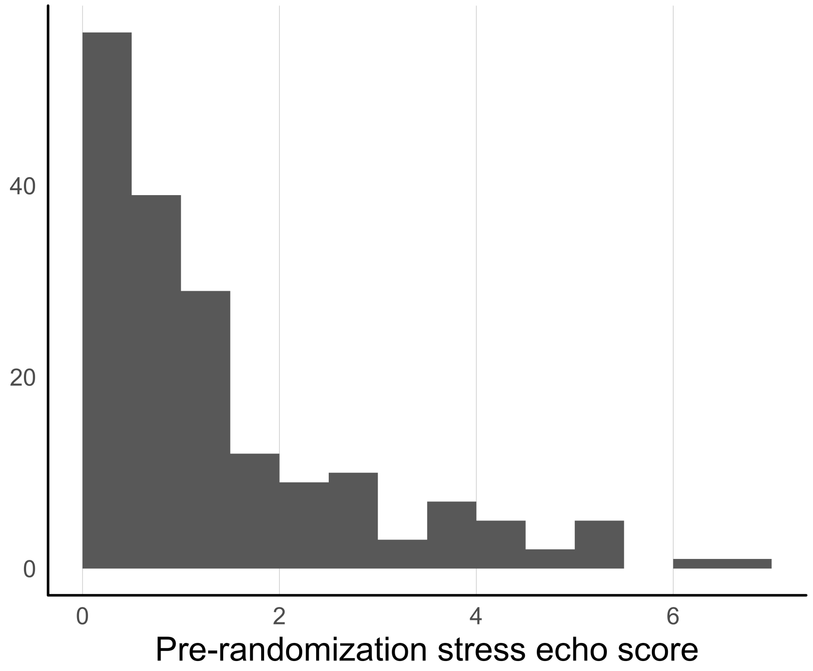

Supplemental Figure 2
Change in stress echo score from pre-randomization to follow-up in 161 patients with stress echo data at both timepoints

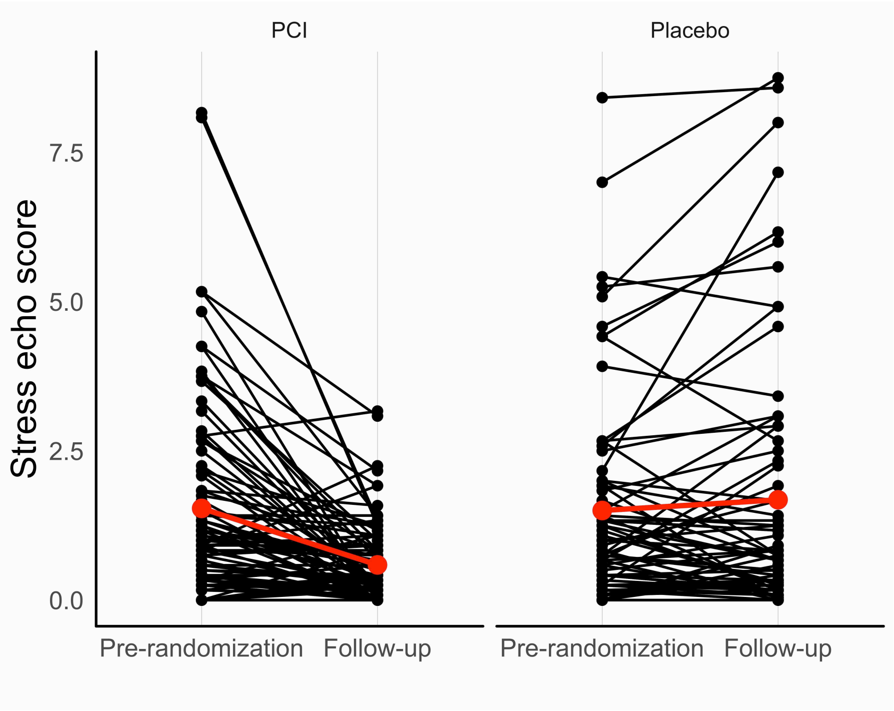

Supplemental Figure 3
Relationship of treatment difference in Seattle Angina Questionnaire quality of life score and pre-randomization stress echo by randomization arm

There is no discernible dependency on pre-randomization stress echo score.

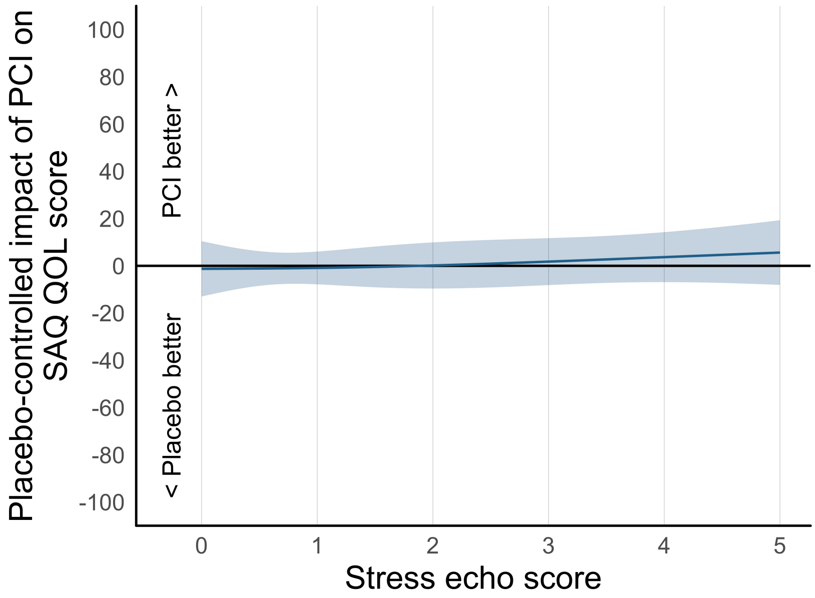

$p_{interaction}$ = 0.689

Supplemental Figure 4
Relationship of treatment difference in EQ-5D-5L quality of life score and pre-randomization stress echo by randomization arm

There is no discernible dependency on pre-randomization stress echo score.

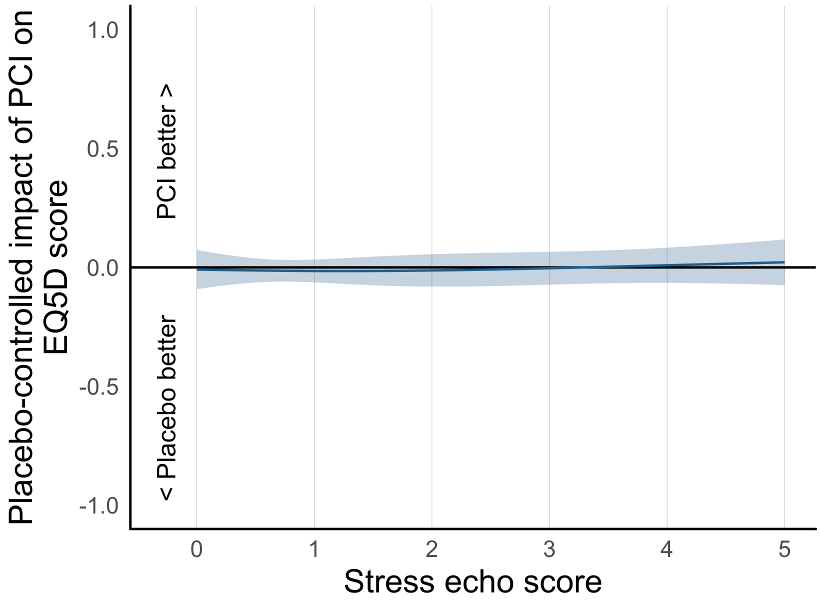

$p_{interaction}$ = 0.789

Supplemental Figure 5
Relationship of treatment difference in CCS class and pre-randomization stress echo by randomization arm

There is no discernible dependency on pre-randomization stress echo score.

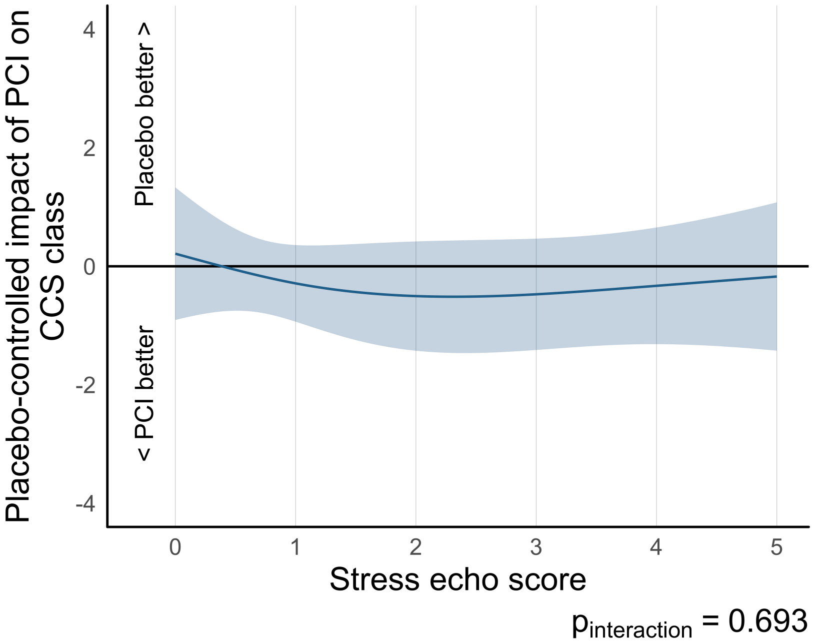

Supplement: Supplementary file 1 [file cir-140-1971-s001.pdf]
